# Patent Foramen Ovale in Cryptogenic Ischemic Stroke: Direct Cause, Risk Factor, or Incidental Finding?

**DOI:** 10.3389/fneur.2020.00567

**Published:** 2020-06-25

**Authors:** Stefanos G. Ioannidis, Panayiotis D. Mitsias

**Affiliations:** ^1^Department of Neurology, University Hospital of Heraklion, Heraklion, Greece; ^2^School of Medicine, University of Crete, Heraklion, Greece; ^3^Department of Neurology and Comprehensive Stroke Center, Henry Ford Hospital, Detroit, MI, United States; ^4^School of Medicine, Wayne State University, Detroit, MI, United States

**Keywords:** ischemic stroke, cryptogenic stroke, patent foramen ovale, atrial septal defect, right-left shunt, paradoxical embolism

## Abstract

Patent foramen ovale (PFO) has been associated with cryptogenic stroke. There is conflicting data and it remains uncertain whether PFO is the direct cause, a risk factor or an incidental finding. Potential stroke mechanisms include paradoxical embolism from a venous clot which traverses the PFO, *in situ* clot formation within the PFO, and atrial arrhythmias due to electrical signaling disruption. Main risk factors linked with PFO-attributable strokes are young age, PFO size, right-to-left shunt degree, PFO morphology, presence of atrial septal aneurysm, intrinsic coagulation-anticoagulation systems imbalance, and co-existence of other atrial abnormalities, such as right atrial septal pouch, Eustachian valve and Chiari's network. These may act independently or synergistically, multiplying the risk of embolic events. The RoPE score, a scale that includes factors such as young age, cortical infarct location and absence of traditional stroke risk factors, is associated with the probability of a PFO being pathogenic and stroke recurrence risk after the index stroke. Multiple investigators have attempted to correlate other PFO features with the risk of PFO-related stroke, but further investigation is needed before any robust conclusions are reached. PFO presence in young patients with cryptogenic stroke should be considered as etiologically suspect. Caution should be exercised in interpreting the relevance of other PFO features.

## Introduction

**Figure 1:**
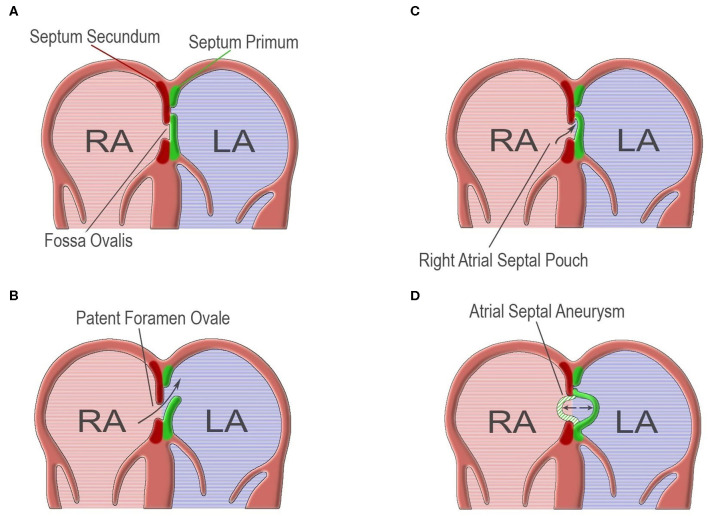
**(A)** Normal atrial septum which results from the fusion of septum primum and septum secundum. **(B)** Failure of fusion of septum primum and septum secundum, leading to patent foramen ovale. **(C)** Right atrial septal pouch, resulting from malformation of atrial septum forming a blind-end socket. **(D)** Atrial septal aneurysm, the result of a hypermobile atrial septum. (Design and courtesy of Mr. Fotis G. Ioannidis).

The atrial septum is formed during the embryogenesis by two membranes growing from the atrial walls (septum primum and septum secundum), leaving an oval shaped fenestration (foramen ovale), which serves the right-to-left shunt (R-L shunt) of the fetal circulation (Figure 1). The foramen ovale is sealed during the first year of life by the fusion of the two membranes. The failure of this process leads to an interatrial slit-like channel, the patent foramen ovale (PFO) (1–3) (Figure 1). PFO is considered to be a subclass of ostium secundum defects (4). Other atrial septal defects include ostium primum defects, sinus venosus defects and coronary sinus defects. The size and morphology of the defect is individualized, depending on the structures which are involved (4).

PFO is present in ~25% of the general population, tending to decline with increasing age, and is the most frequent cause of R-L shunt in adults (2, 5–7). Although most of the times PFO is “innocent,” it has been associated with cryptogenic stroke (CS), migraine, peripheral embolism, and Alzheimer's dementia (1). The link between PFO and stroke was first described by Cohnheim in 1877 (8), and since then, a strong association has been established. The high PFO prevalence in the CS population (about 50%, 2-fold when compared with stroke patients of known cause) cannot be overlooked (5, 9).

The population affected by PFO-related embolic events is mostly young, and although the annual recurrence risk is relatively low, it tends to aggregate to a non-negligible total rate (6, 10). On the other hand, many PFOs in stroke patients represent incidental findings (11). Thus, it is essential to determine the high risk features of PFOs, as only PFO-related CS patients will potentially benefit from a PFO-closure procedure (6, 12, 13).

## PFO and Stroke

CS comprises 15–40% of all ischemic strokes, and PFO occurs in 40–56% in patients <55 years old with CS or transient ischemic attack (TIA) (6, 12, 14). One has to distinguish between PFO being a direct cause of stroke and PFO being a risk factor for stroke. The relevant literature indicates that the strength of the association between PFO and stroke depends on the type of study. The role of PFO as a risk factor for ischemic stroke has mainly been demonstrated in case-control studies. In one of the original case-control studies, an ~4-fold increase in PFO prevalence in stroke patients younger than 55 years and an ~2-fold increase in older patients compared with controls of similar age was demonstrated (15). In a robust meta-analysis of case-control studies, Overell et al. reported an OR of 3.1 for PFO, 6.14 for atrial septal aneurysm (ASA), and 15.59 for PFO combined with ASA, when the examined population was younger than 55 years (16). On the contrary, the role of PFO as a risk factor for stroke and vascular events in the general population has not been demonstrated with certainty. Most studies suffered from inadequate sample sizes or short follow-up durations which may have masked possible associations. Di Tullio et al. (17) reported the results of a population study in which they followed a community cohort of asymptomatic individuals with and without PFO for an average of 11 years, and demonstrated that PFO was not associated with an increased risk of clinical stroke or silent brain infarcts (17).

Some PFOs likely are incidental findings. When they are pathogenic, it is still debatable whether they represent a risk factor for stroke or the true cause (5, 6). Moreover, the precise mechanism by which PFO causes a stroke is uncertain. Several PFO characteristics have been reported as high-risk features, such as hypermobile atrial septum, R-L shunt grade, R-L shunt at rest, as well as non-PFO features, as young age and the coexistence of other atrial septal abnormalities (1, 16, 18).

## Potential Stroke Mechanisms in PFO

### Paradoxical Embolism

The most acceptable hypothesis currently is that of paradoxical embolism (19, 20). This phenomenon requires a venous thrombus to travel through a R-L shunt and cause arterial embolism (5, 6). This hypothesis is supported by studies reporting the PFO size and R-L shunt grade as risk factor for CS, case reports of thrombi stuck in PFO tunnel, and CS following deep venous thrombosis (DVT) (5, 19).

However, paradoxical embolism cannot stand as the only possible explanation (21). The existing data do not support an increased incidence of DVT or Valsalva-like activities prior to CS as compared to non-PFO CS patients, and a venous source of embolism is rarely identified (22). Moreover, some studies report increased risk of recurrence associated with smaller shunts (13). Thus, additional or alternative explanations are in order, perhaps related to PFO characteristics (18).

### *In situ* Clot Formation

Accumulated data support the notion that PFO is liable for *in situ* thrombus formation (13, 20–22). This hypothesis is empowered by the fact that specific features, such as long-tunneled PFO, concomitant presence of ASA or Chiari's network, increase the risk of stroke (1, 23–25). These findings do not favor the paradoxical embolism hypothesis, but the deceleration of flow, blood stagnation and thrombi formation within the PFO or ASA (20, 26).

Rigatteli et al. reported observations from computational anatomical models where he noted a pathologic pattern of left atrial (LA) blood flow due to permanent R-L shunt (27). Furthermore, a prospective study comparing pre-closure PFO patients with atrial fibrillation (AF) patients and healthy individuals, claimed that moderate-to-severe ASA was correlated with LA dysfunction (active and passive emptying, conduit function, LA ejection fraction), which reversed after PFO closure (28). These very interesting findings suggest LA dysfunction and AF-like flow, forming thrombi in the absence of the arrhythmia. Moreover, LA size has been correlated with ASA presence, multiple ischemic lesions and the RSL degree; LA diameter ≥43 mm and RoPE score>7 were significantly associated (29). Questions are also raised regarding the involvement of other R-L shunt sites (25).

### Arrhythmias

A very attractive hypothesis, supported by several authors, claims that embolic events in PFO are caused by atrial tachyarrhythmias and/or paroxysmal AF, especially in the presence of a hypermobile atrial septum (22, 30–33). Indeed, 20–42% of PFO and/or ASA patients are considered to have AF or atrial flutter (31).

The term of atrial vulnerability describes the electrophysiological trend to induce AF. Berthet et al. reported that inducible AF longer than 60 s in duration and abnormalities of effective refractory periods and atrial conduction time, were present in 58% of patients with PFO and/or ASA, as compared to 25% of patients without (31). Moreover, Cotter et al. reported increased interatrial block and atrial vulnerability in young CS patients with PFO; cases were also found to have longer P-wave duration, and proposed that stretch or pressure on the atrial septum is the causative mechanism (34).

It is believed that each one of the above mechanisms exists and that their synergistic action results in cumulative outcomes.

## Age

Several studies support that one of the most powerful markers of a non-incidental PFO in stroke patients is young age, usually defined as age ≤ 55 years (1, 16, 35, 36). The incidence of PFO in the stroke population tends to decrease with increasing age (0–30 years: 34.3%, 30–80 years: 25.4%, 90–100 years: 20.2%), while other more traditional stroke risk factors, such as hypertension, dyslipidemia, smoking, and arrhythmias increase (2, 5, 22). The latter factors are also less frequent in populations with PFO-attributable embolic events (22).

In a meta-analysis, Overell et al. reviewed the literature with an eye toward the three-way association between PFO, CS and age heterogeneity of study populations, and concluded that when older patients were included, the strength of the correlation between PFO and CS was rather low (16). Specifically, when comparing stroke patients with controls, the positive association of PFO with CS was a function of younger age of the population (mean age of 44.8 years), while in the older population (mean age of 61.1 years) this association was not present (16). A similar pattern was detected when comparing CS to patients with stroke of known cause or healthy individuals (16, 35). Another meta-analysis reported similar findings, with OR of 5.1 for association of PFO with CS in young patients, while the association was weaker (OR: 2.0) for patients older than 55 years (36). According to these data, the presence of PFO in young CS patients should be strongly considered as etiologically suspect (16).

Nevertheless, it is worth noting that PFO-attributable strokes do occur in older patients as well, although data are scarce and further investigation is needed (24, 37). The population-based study of Mazzucco et al. is in line with this statement, and suggests transcranial Doppler testing as a feasible and cost-effective screening (38).

## High-Risk Anatomical Features of PFO

### Size

PFO diameter ranges from 1 to 19 mm, and tends to grow larger with advancing age (1, 2). Although PFO diameter is well established as a risk factor, the existing data are conflicting due to inter-operator variability and differences in the estimation methodology. It is worth mentioning here that the number of microbubbles crossing the atrial septum is not a reliable way for assessing the anatomic size of the PFO (18).

In most studies, size is an independent risk factor for stroke occurrence and recurrence (1, 5, 39, 40), with OR of 2.54 when the size is ≥2 mm (41). Moreover, CS patients tend to have larger PFOs, when compared to stroke patients of other known causes (13, 36). The impact of size on TIAs seems to be weaker (11).

On the other hand, some studies demonstrated that large PFOs were associated with increased risk for the index event or its severity, while smaller PFOs were associated with the risk of recurrence, indicating different pathophysiological mechanisms of embolism (13, 14).

### Shunt Degree

PFO may prevent shunting if its morphology favors a sufficient valvular mechanism; otherwise, it allows a shunt of varying degree (1, 3). The shunt is best estimated by transesophageal echocardiography. Transcranial doppler testing is highly sensitive but detects any R-L shunt, which includes intracardiac and extracardiac locations (1, 5). As for the transthoracic echocardiography, it is believed that it is more specific but less sensitive in detecting PFO, in comparison to transcranial Doppler ultrasonography (42).

Shunt degree is not defined exclusively by PFO size; (11) on the contrary, the right-left atrial pressure difference is one of the main factors affecting the degree of the shunt. For example, pulmonary hypertension favors patency of foramen ovale (2), while mitral regurgitation, left atrial dilatation, and left ventricular hypertrophy can raise the LA pressure and diminish the R-L shunt degree (43).

R-L shunt can be detected in up to 100% of patients with PFO and history of embolism; 10% of PFO-related CS have large-degree R-L shunt (44, 45). The shunt degree is significantly associated with stroke risk (both for index or recurrent event), as well as with TIA and migraines, while asymptomatic PFOs tend to be smaller (1, 9, 25, 36, 39, 41, 43). The incidence of stroke may be higher in the presence of significant shunt at rest (1). Moreover, smaller R-L shunts have been associated with greater recurrence risk (1, 13, 35, 46). It is also interesting that echocardiography features may predict recurrence risk only in those patients with higher RoPE scores (for RoPE score analysis, please, see below) (13). It has been suggested that when the RoPE score is ≥7 the presence of hypermobile interatrial septum and smaller shunts are predictive of stroke recurrence; if these data is confirmed, then we can consider that paradoxical embolism is responsible for only a fraction of the PFO-associated strokes, and that additional potential pathogenic mechanisms exist (13).

Interestingly, some studies report that the degree of R-L shunt is similar in PFO-related and other etiology stroke patients, and that it is not linked with risk of recurrence (18, 20). Nevertheless, one should keep in mind that shunt degree is a dynamic variable which can change because of pressure changes in the cardiac chambers, patient cooperation during the exam and operator's skills, indicating that its reliability and significance in clinical practice may be limited (18, 47). Moreover, the variability and controversies in the existing literature can be explained by the differences of the definitions of degree of R-L shunt and also of the population under study.

### Morphology

Other potentially high-risk features of PFO are: PFO length, tunnel-like morphology, height, thick fossa ovale rims, and low-angle PFO (14, 35, 41). Unfortunately, data are scarce, usually are the result of rather small studies, and often are conflicting.

One of the high-risk characteristics is the distance between septum primum and septum secundum, often named “PFO height.” Some studies demonstrated increased embolic risk when the separation of the two membranes is large. Other studies report increased risk when the overlap between septum primum and septum secundum, often named “PFO length,” is deficient (25, 35, 43). Tunnel-like morphology, defined as ≥8–10 mm in length, was also reported as a high-risk factor, with OR for CS in the region of 2.66 (*p* = 0.017) (1, 25, 35, 41). The discrepancy of whether a longer or shorter PFO is associated with embolic events may indicate differences in pathogenetic mechanisms.

Although the thickness of fossa ovale rims has not been linked with definite embolic risk, excessive thickness can be associated with poor closure devise stability (35).

Finally, the angle of PFO in relation to the inferior vena cava has been associated with the embolic risk. More specifically, a low-angle PFO (≤10°) corresponds to OR 3.74 (*p* = 0.029) for CS (41).

The above statements are made with a sense of caution as other studies failed to confirm these results (48).

## Atrial Septal Aneurysm

Atrial septal aneurysm (ASA) is an excursion of a hypermobile interatrial septum, which floats to either direction in the atria, and involves septum segments of variable size (5, 33) (Figure 1). Wide heterogeneity exists in the literature because of differences in the definition of ASA and study populations (16). The prevalence of ASA in the general population is 1–4% (15). Usually, ASA is combined with PFO (60–89%), and when it does, PFO tends to be of larger size (20, 40, 49). Several other abnormalities have been correlated with ASA, such as atrial septal defects and mitral valve prolapse (24, 33).

ASA is associated with increased stroke risk, especially in the presence of PFO, and is considered a stronger risk factor than PFO (5, 16, 24, 41, 50, 51). The incidence is even higher in younger patients and those with PFO-attributable stroke (16, 52). Moreover, atrial septal hypermobility has been identified as an independent predictor of embolism recurrence, and the risk rises by two to three times when it coexists with PFO (1, 11, 39, 46). Interestingly, the risk of recurrence for stroke or TIA within 4 years after the initial event was estimated at 19.2% for PFO combined with ASA vs. 5.6% for PFO alone (20). Furthermore, when PFO and ASA co-existed the OR for stroke was 4.96, compared with 1.83 for PFO or 2.35 for ASA in isolation (16). The risks seem to apply to older patients as well (37).

Besides the synergistic action of PFO and ASA, there is a size-dependent effect of ASA on stroke risk. Cabanes et al. reported that in young patients the OR for stroke was 8.5 when the ASA excursion was >10 mm, and only 1.2 for excursion 6–10 mm (26). A small study comparing symptomatic and asymptomatic ASA reported median excursion of 7 mm in the patients group, in contrast to 4 mm in the healthy individuals group (14). Similar differences were also found in other studies, but these findings needs further investigation and validation (11).

It is important to mention here that in the general population ASA is associated with increased stroke risk, but the relative risk is still low, and therefore screening tests for asymptomatic individuals are not recommended (15).

## Other Atrial Abnormalities

Several other atrial structural abnormalities have been considered to be associated with PFO and increased embolic risk, such as right atrial septal pouch (RASP), prominent Eustachian valve or ridge and Chiari's network (1, 23).

### Right Atrial Septal Pouch

Right atrial septal pouch (RASP) is a sack-shaped atrial septal malformation, detected on either side of the septum (1) (Figure 1). Scarce data propose RASP as a cause of blood flow disturbance and embolus formation, and there is coexistence with PFO arterial embolic events may occur (1).

### Eustachian Valve and Chiari's Network

Eustachian valve and Chiari's network are fetal features that interfere with the normal embryonic R-L shunt (1). Eustachian valve co-occurrence with PFO is estimated at 70%, while Chiari's network is related with PFO in 83% of cases (5).

While both can represent incidental findings, they have also been recognized as stroke risk co-factors in the presence of PFO (25, 40, 41). In particular, in a retrospective study, the OR for Eustachian valve or Chiari's network as factors related to CS was 4.47 in univariate analysis (*p* = 0.002) and 4.71 in multivariate analysis (*p* = 0.009) (41).

### Hybrid Defects

The term “hybrid defects” refers to a group of heterogeneous atrial septal abnormalities associated with PFO. (1) These combinations include ostium primum, ostium secundum, sinus venosus, and coronary sinus defects (5). Theoretically, all may result in paradoxical embolism, but their exact role and stroke risk associated with them still remain undetermined.

## Venous Thrombosis

Because paradoxical embolism is considered as one of the main mechanisms of PFO-related stroke, a clot in the venous system or conditions predisposing to venous clots are usually sought for. Deep venous thrombosis (DVT), pelvic vein thrombosis and hypercoagulable states are considered as risk factors for PFO-related stroke (35, 40).

In a rather small study increased incidence of lower extremity DVT was found in patients with probable paradoxical embolism (53). Similar findings were reported in a study of a young CS population (54). Moreover, DVT was associated with strokes >3 cm in diameter (55). Conditions such as immobilization, anesthesia, surgery and pregnancy prior to stroke events were found more often in CS patients with PFO (4.5 vs. 1.6%, *p* = 0.05) (22). However, other studies are not in line with these data, and claim that the source of venous thrombi is rarely detected (11, 40, 56). Of course, the discrepancy in the frequency of DVT and the usually low frequency of identifiable DVT among studies addressing PFO-related stroke may be in part due to the late timing of the diagnostic studies of the venous system after the index stroke.

Disruption of the balance of natural coagulation-anticoagulation mechanisms, such as Factor V Leiden mutation or prothrombin gene mutation, is also a co-factor for increased risk of stroke in the presence of R-L shunt. Karttunen et al. report OR 2.8 (*p* = 0.021) for prothrombotic states and 2.5 (*p* = 0.037) for common risk factors for venous thrombosis, in a case-control study of CS in PFO patients, aged 15–60 years (57). An underlying thrombophilia, either inherited or acquired, also predisposes to recurrence of embolism; this risk is decreased with PFO closure (58).

The risk of formation of venous clots seems to interact with age, as older people have more risk factors leading to this process. In the presence of a PFO, paradoxical embolism may occur, and recurrence rates tend to be higher (37, 59).

Of interest is the results of two clinical trials addressing the question of treatment with antiplatelet vs. anticoagulant drugs for second stroke prevention in patients with CS and underlying PFO. In the PICSS (patient foramen ovale in cryptogenic stroke study), a substudy to the WARSS (warfarin vs. aspirin for recurrent stroke study), there was no significant superiority of warfarin anticoagulation over aspirin; there was however a trend of toward warfarin being better than aspirin for secondary stroke prevention in this setting (HR = 0.52, *p* = 0.28); it should be noted that the follow-up period was 2 years (60). From the CLOSE trial, Mas et al. demonstrated that anticoagulants were not superior vs. aspirin for stroke prevention; this arm of the trial was underpowered (61). Based on the above and the knowledge that anticoagulants are the main treatment for venous thromboembolism, the lack of solid evidence that anticoagulants perform better than antiplatelet agents in preventing stroke, despite the methodological problems for each study, could raise suspicion that paradoxical embolism may not be the main or most frequent mechanism of stroke causation in the setting of PFO. Further study on this matter is desperately needed.

## The Rope Score and PFO as an Incidential Finding

Many authors have attempted to answer which features of a PFO determine whether it is pathogenic or incidental finding in CS patients.

The Risk of Paradoxical Embolism (RoPE) score was designed for this reason, and also estimates the recurrence risk within 2 years after the index event (7). RoPE scale components include age, hypertension, diabetes mellitus, stroke or TIA history, smoking, and neuroimaging (large cortical infarct) to determine a 10-point score (7). Higher RoPE score results from young age, cortical infarcts and the absence of traditional stroke risk factors; the higher the RoPE score the more likely that a PFO is pathogenic, and is usually associated with lower risk of stroke recurrence (7). RoPE score of 0–3 estimates 0% probability of pathogenic PFO and 20% probability of recurrent event, while a score of 9-10 estimates 88 and 2% probability, respectively (7). As emphasized above, PFO-attributable strokes may have low recurrence risk within the short period of 2 years, but because they occur in young patients, the overall risk within the lifespan of patients may be verysubstantial (6). It is worth noticing that R-L shunt degree, ASA and other PFO high-risk characteristics were not included in the RoPE score variables (47). Furthermore, in cases of stroke of known etiology, the RoPE score loses its prognostic value (7).

The RoPE score is a probability index; thus, low scores cannot exclude with certainty the possibility of PFO-attributable stroke, while higher scores cannot confirm the causative relationship (7, 25). Nevertheless, its efficacy has been tested in clinical practice; the fact that the risk of stroke recurrence was still high after PFO closure in patients with low RoPE score indicates that the stroke mechanism was indeed unrelated to PFO (62). A study in CS patients ≤ 50 years reported that RoPE score above 7 is the optimal limit for identifying a causative relationship of PFO and CS (63). It should be emphasized though, that the RoPE score does not characterize the risk of stroke associated with PFO individually, but it rather provides a guide to define whether the relationship of PFO with CS after the index event is causative or not (6).

Other high-risk echocardiographic features should not be underestimated. Recurrence risk seems to be heterogeneous within each RoPE score strata. Thaler et al. report an increased recurrence risk in patients with high RoPE score, associated with history of stroke or TIA, hypermobile atrial septum and small R-L shunt (13). Moreover, a meta-analysis defined that in the co-existence of ASA the probability of a PFO to be incidental was decreased (9% in younger and 26% in older patients) (36). The same study reported that when using the Bayesian approach one third of all PFOs in CS patients are incidental, and morphologic characteristics may alter these rates (36). A very interesting retrospective cohort study attempted to associate high-risk morphological features of PFO with the probability of CS (41). They identified: (a) long-tunnel PFO ≥10 mm, (b) hypermobile interatrial septum, (c) Eustachian valve/Chiari's network, (d) large R-L shunt during Valsalva maneuver, and (e) low-angle PFO ≤ 10°, as high-risk echocardiographic features and assigned one point to each creating thus a 5-point scale. PFO associated with a score ≥2 in this scale was strongly linked with CS (41). This study had several limitations, but it sets the basis for further investigation.

## Conclusions

PFO in stroke patients may represent an incidental finding, a risk factor for stroke occurrence or a robust cause. It is associated with CS through several mechanisms; most theories support paradoxical embolism, *in situ* thrombus formation, and arrhythmogenesis, while other possible, yet unknown, explanations cannot be excluded. Young age, PFO morphological characteristics and factors predisposing to venous thrombosis are essential features to determine a pathogenic PFO. Further investigation is needed in order to identify the role of these characteristics in the stroke pathogenesis.

## Author Contributions

SI: review of literature, writing of manuscript draft, review of final draft manuscript. PM: review of literature, critical review of final draft of manuscript. All authors contributed to the article and approved the submitted version.

## Conflict of Interest

The authors declare that the research was conducted in the absence of any commercial or financial relationships that could be construed as a potential conflict of interest.
